# Sirolimus for Secondary Prevention of Cutaneous Squamous Cell Carcinoma in Kidney Transplant Recipients: A Systematic Review and Meta‐Analysis of Randomized Controlled Trials

**DOI:** 10.1111/ijd.70285

**Published:** 2026-01-17

**Authors:** Yannick Foerster, Julia Palaras, Kristine Mayer, Tilo Biedermann, Oana‐Diana Persa

**Affiliations:** ^1^ Department of Dermatology TUM School of Medicine and Health Munich Germany; ^2^ Institute of Medical Biostatistics, Epidemiology and Informatics Mainz Germany

**Keywords:** cSCC, kidney transplant, sirolimus, skin cancer, squamous cell carcinoma

## Abstract

Kidney transplant recipients (KTRs) are at increased risk of developing cutaneous squamous cell carcinoma (cSCC), particularly when treated with calcineurin inhibitors (CNI), which are strongly associated with tumorigenesis. In contrast, mTOR inhibitors such as sirolimus have demonstrated antitumor activity, but their role in secondary prevention of cSCC remains unclear. We conducted a systematic review and meta‐analysis of randomized controlled trials (RCTs) to evaluate the impact of switching from a CNI‐based to an mTOR inhibitor‐based immunosuppressive regimen on the incidence of cSCC in KTRs with prior cSCC. MEDLINE, EMBASE, CENTRAL, and trial registries were searched through June 2025. Incidence rate ratios (IRRs) for cSCC and risk ratios (RRs) for adverse events (AEs) were pooled using a random‐effects model. Risk of bias was assessed with Cochrane RoB2. The study was registered in PROSPERO prior to data extraction (CRD42024583966). The study was unfunded. Four RCTs (393 patients) were included. Sirolimus significantly reduced 2‐year cSCC incidence (IRR 0.51, 95% CI 0.39–0.67). However, discontinuation was more frequent (RR 8.60, 95% CI 1.95–37.93) due to AEs. No significant differences in mortality or graft rejection were found. Certainty of evidence was high for cSCC incidence and low for adverse events due to heterogeneity and selective reporting. In conclusion, sirolimus reduces secondary cSCC risk but increases AEs; patient selection and monitoring are essential.

**Trial Registration:** PROSPERO number: CRD42024583966

## Key Points

- Why was the study undertaken?
  ○ Kidney transplant recipients (KTRs) are at high risk for developing cutaneous squamous cell carcinoma (cSCC), and the risk dramatically increases after a first cSCC.
  ○ Immunosuppressive therapy with calcineurin inhibitors (CNIs) is considered largely responsible for the high tumorigenicity due to reduced immune surveillance and direct oncogenic effects.
  ○ In contrast, mTOR inhibitors such as sirolimus have demonstrated antitumor properties.
  ○ This study was conducted to evaluate whether switching immunosuppressive therapy to sirolimus can reduce the risk for secondary cSCCs in KTRs.
- What does this study add?
  ○ Switching from CNIs to mTOR inhibitors is effective for primary prevention of skin cancer in KTRs.
  ○ Routine conversion of all patients is not feasible due to a higher rate of side effects. Balancing the tumor‐reducing benefits of sirolimus with its side effect profile is critical for optimal patient management.
  ○ This study provides new evidence specifically focused on secondary prevention, showing that switching to sirolimus significantly reduces the risk of subsequent cSCC in high‐risk patients with prior cSCC.
- What are the implications of this study for disease understanding and/or clinical care?
  ○ The study provides evidence‐based support for a targeted conversion strategy in KTRs with a history of cSCC.
  ○ It introduces secondary prevention as a clinically meaningful approach for reducing cancer burden in high‐risk transplant patients.
  ○ This shifts the focus from blanket prevention to personalized, risk‐adapted immunosuppression management, potentially improving patient outcomes while minimizing unnecessary side effects.

## Graphical abstract

Meta‐analysis of randomized controlled trials shows that switching from calcineurin inhibitors (CNI) to sirolimus in kidney transplant recipients (KTRs) with prior cutaneous squamous cell carcinoma (cSCC) reduces secondary cSCC incidence but increases adverse‐event–related treatment discontinuation.

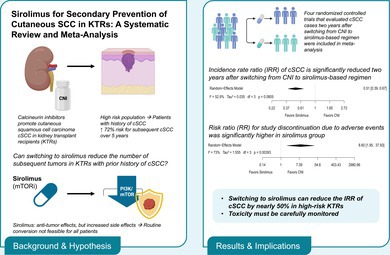

## 1 Introduction

Cutaneous squamous cell carcinoma (cSCC) is the most common malignancy in kidney transplant recipients (KTRs), occurring up to 250 times more frequently compared to the general population [1, 2, 3]. Calcineurin inhibitors (CNIs) remain the standard immunosuppressive therapy and are recommended by international guidelines [4]. However, long‐term therapy with CNI is associated with an increased risk for cSCC due to their effects on immune surveillance and tumor promotion [5].

In contrast to CNI, mTOR inhibitors such as sirolimus have demonstrated significant antitumor activity [6, 7]. A prior meta‐analysis has shown that switching from CNI to sirolimus is beneficial for primary prevention of skin cancer in KTRs [8]. However, because mTOR inhibitors are associated with a higher incidence of adverse events and treatment discontinuation, routine conversion of all patients is not reasonable in clinical practice [9, 10]. Also, current evidence suggests that KTRs with a history of cSCC are at particularly high risk for developing subsequent tumors, and studies have shown a 1‐year risk of 32% for a second skin cancer, increasing to 72% at 5 years [11, 12].

Early detection and treatment of new cSCC lesions is important in these patients because they have a higher risk of metastatic progression compared to the immunocompetent population [13, 14]. Once metastatic or locally advanced disease occurs, systemic therapy with checkpoint inhibitors is associated with a high risk of allograft rejection in KTRs, significantly limiting treatment options [15, 16, 17, 18].

Therefore, identifying effective strategies for secondary prevention of cSCC in this high‐risk group is critical. In this context, we conducted a systematic review and meta‐analysis of randomized controlled trials (RCTs) to evaluate the impact of switching from a CNI‐based to an mTOR inhibitor‐based immunosuppressive regimen on the incidence of cSCC in KTRs with prior cSCC.

## 2 Materials and Methods

### 2.1 Protocol and Registration

This review was prospectively registered in the International Prospective Register of Systematic Reviews prior to data extraction. The study was conducted in accordance with the Preferred Reporting Items for Systematic Reviews and Meta‐Analyses (PRISMA) guidelines, the Cochrane Handbook for Systematic Reviews, and the Enhancing Transparency in Reporting the Synthesis of Qualitative Research (ENTREQ) statement.

### 2.2 Eligibility Criteria

The eligibility criteria were defined in accordance with the population‐intervention‐comparison‐outcomes framework (PICO).

**Population:** All kidney transplant recipients with a history of at least one cSCC who were receiving standard CNI‐based immunosuppressive therapy were eligible for inclusion.

**Intervention:** The intervention was defined as the transition from standard CNI‐based immunosuppressive therapy to a sirolimus‐based regimen.

**Comparison:** Patients who continued standard CNI‐based immunosuppressive therapy served as the comparison group.

**Outcomes:** Incidence of cSCC at 2 and 5 years after switching immunosuppressive therapy. Also reported adverse events over 2 years were evaluated.

### 2.3 Information Sources and Search Strategy

The electronic databases MEDLINE, EMBASE, and the Cochrane Central Register of Controlled Trials (CENTRAL) were systematically searched from database inception through June 27, 2025. Additionally, the EU Clinical Trials Register (https://www.clinicaltrialsregister.eu), Australian New Zealand Clinical Trials Registry (https://www.anzctr.org.au), and US National Institutes of Health Clinical Trials Register (https://clinicaltrials.gov) were manually screened. The complete search strategy is detailed in Table S1.

### 2.4 Study Selection and Data Collection

Two reviewers (YF and OP) independently screened all records identified through Ovid, a web‐based search platform. Records were initially screened by title and abstract and then by full‐text review according to predefined eligibility criteria. The same investigators also searched relevant clinical trial registries. Any discrepancies were resolved by consensus.

Only RCTs were eligible for inclusion. No language restrictions were set.

### 2.5 Data Synthesis

To quantify the effect sizes, a meta‐analysis of incidence rate ratios (IRRs) was conducted. IRRs were chosen because they incorporate person‐time as the denominator and therefore account for all events, including recurrent tumors within the same individuals. In addition, IRRs more appropriately accommodate differences in follow‐up duration resulting from study discontinuation rates. For each included study, the number of cSCC cases and the corresponding person‐years of follow‐up were extracted for both treatment groups (Sirolimus vs. CNI). Effect sizes and variances were calculated using the escalc function from the metafor package (Version 4.8‐0) for RStudio (Version 4.4.2). A random‐effects analysis was performed using the restricted maximum‐likelihood estimator (REML). A forest plot was generated to visualize individual and pooled effect estimates, including the percentage weight of each study and measures of heterogeneity (Tau^2^, *I* ^2^, and the *Q*‐test for heterogeneity). In addition, adverse events were assessed using risk ratios (RRs) as a binary first‐event measure. For this analysis, the number of events and total participants in both intervention and control groups were extracted. Effect estimates and standard errors were calculated using the escalc function. A separate random‐effects meta‐analysis was performed to evaluate the risk of adverse events associated with Sirolimus compared to CNI treatment.

### 2.6 Risk of Bias Assessment and Certainty of Evidence

Two authors (YF and OP) independently assessed the risk of bias for all included studies using the Revised Cochrane Risk of Bias Tool for Randomized Controlled Trials (RoB2). Any discrepancies were resolved through discussion and consensus. If at least ten RCTs were included, publication bias was planned to be evaluated using a funnel plot and Egger's test. To assess the robustness of the results, a sensitivity analysis was conducted by sequentially omitting individual studies. Certainty of evidence was rated according to the GRADE (Grading of Recommendations Assessment, Development and Evaluation) approach.

## 3 Results

### 3.1 Study Identification

**FIGURE 1:**
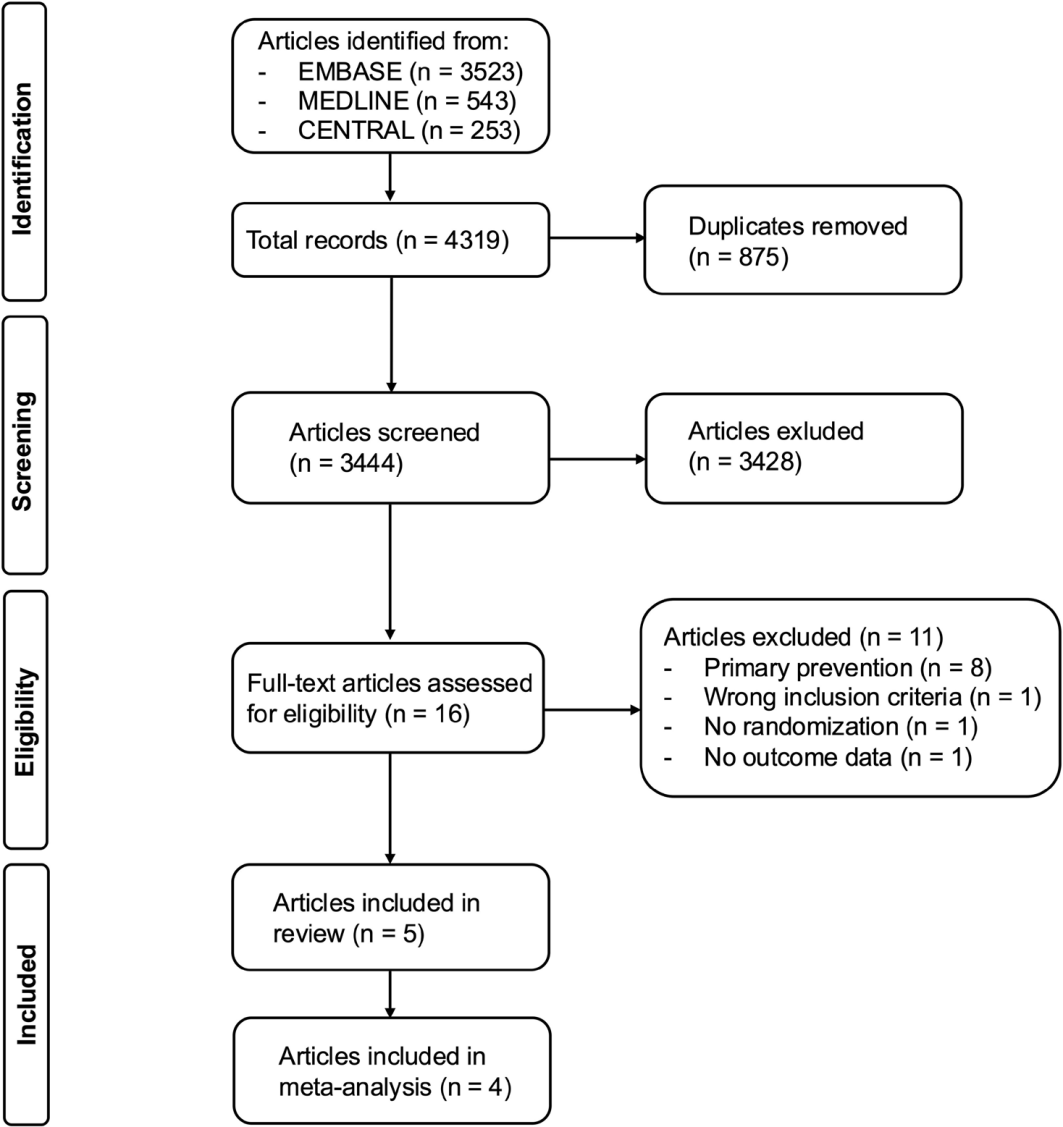
Flowchart of the study selection process according to the Preferred Reporting Items for Systematic Reviews and Meta‐Analyses (PRISMA). A total of 4319 studies were identified from the electronic databases EMBASE, MEDLINE and CENTRAL. After removing duplicates, 3444 articles were screened for eligibility, and 16 underwent full‐text review. Five studies were included in our review, and four were used for quantitative data synthesis.

Our literature search identified 4319 records. After removing duplicates and screening titles and abstracts, 16 records were selected for full‐text review. Of these, eight records were excluded because they focused on primary prevention [19, 20, 21, 22, 23, 24, 25, 26]; one record was excluded because it did not distinguish between cSCC and other skin lesions [27], including verrucae; one record lacked a randomized design [28] and one study was terminated early without available outcome data [29]. Five studies were included in our review and four studies were included in the quantitative synthesis of results (Figure 1). Details of the included studies are summarized in Table 1 and a brief overview of studies excluded after full‐text review is provided in Table S2.

**TABLE 1:** Summary of included articles.

Author	Year	Country	Description	Risk features
Hoogendijk‐van den Akker et al. [30]	2023	Netherlands, UK	155 patients included, 74 in sirolimus and 81 in CNI group Age distribution: Sirolimus group, 31 < 55 years, 43 ≥ 55; CNI group, 23 < 55 years; 58 ≥ 55 Sex distribution (f/m): Sirolimus group, 32/42; CNI group 17/64 Outcome: cSCC rate over 2 years follow‐up	Number of prior cSCCs (one/multiple): Sirolimus group, 30/44; CNI group 23/58 Minimum IS exposure for 12 weeks Skin type (I–III/IV): Sirolimus group, 74/0; CNI group, 78/3
Euvrard et al. [31]	2012	France, Switzerland, Spain	Phase 3 trial (TUMORAPA) 120 patients included, 64 in sirolimus group and 56 in CNI group Age distribution: Sirolimus group, median age 62 (range 37–84); CNI group median age 63 (range 30–76) Sex distribution (f/m): Sirolimus group, 17/47; CNI group 11/45 Outcome: cSCC and skin tumors other than cSCC (BD, KA, BCC, PK) rates over 2 years follow‐up	Number of prior cSCCs (one/multiple): Sirolimus group, 35/29; CNI group 31/25 No minimum IS exposure (Median): Sirolimus group, 148.6 m; CNI group 142.9 m Skin type (I–III/IV): Sirolimus group, 47/17; CNI group, 46/10
Campbell et al. [32]	2012	Australia, New Zealand, US	86 patients included, 39 in sirolimus and 47 in CNI group Age distribution: Sirolimus group, mean age 59.1 years (SE 1.5), CNI group 59 (SE 1.2) Sex distribution (f/m): Sirolimus group 8/31; CNI group 13/34 Included also patients with BCC Outcome: cSCC and BCC rates over 2 years follow‐up	Number of cSCCs in previous 12 months (Mean): Sirolimus group, 2.0 (SD 2.3); CNI group 1.7 (SD 1.8) Minimum IS exposure for 1 year Race (White/Asian): Sirolimus group, 38/1; CNI group, 47/0
Carroll et al. [33]	2013	UK	32 patients included, 13 in sirolimus group and 19 in CNI group Age distribution: Sirolimus group, median age 64 (range 46–72), CNI croup 59 (range 47–81) Sex distribution (f/m): Sirolimus group 4/9; CNI group 1/18 cSCC rates over 2 years follow‐up	Number of previous cSCCs (median): Sirolimus group, 2.0 (range 1–26); CNI group, 3 (range 1–9) No minimum IS exposure (Median): Sirolimus group, 21 years; CNI group 17 years Skin type of all patients was classified as white
Dantal et al. [34]	2018	France, Switzerland, Spain	5 years extention of TUMORAPA study	

*Note:* Five articles were included in our systematic review, and the first four studies were included in the quantitative synthesis. All studies were randomized controlled trials (RCTs).

Abbreviations: BCC, basal cell carcinoma; BD, Bowen's disease; CNI, calcineurin inhibitor; cSCC, cutaneous squamous cell carcinoma; f, female; IS, immunosuppressant; KA, keratoacanthoma; m, male; PK, premalignant keratoses; SD, standard deviation; SE, standard error.

### 3.2 Baseline Risk Characteristics of Included Populations

The included RCTs exhibited notable clinical heterogeneity in baseline characteristics relevant to cSCC risk (Table 1). Prior cSCC burden differed across studies, with one trial enrolling predominantly patients with multiple tumors [30] and one trial including a mix of single and multiple prior lesions [31]. The other two trials only reported measures of central tendency and dispersion for previous tumors [32, 33]. Similarly, cumulative immunosuppressant (IS) exposure varied, and the reporting was heterogeneous. Some studies provided cumulative IS duration [31, 33], while others reported only a minimum required duration of therapy [30, 32]. Participant skin type or race also differed, with most studies including predominantly light‐skinned patients but with variable representation of darker phototypes.

### 3.3 Incidence of Cutaneous Squamous Cell Carcinoma

Four RCTs including 393 patients reported data on cSCC cases within two years following the switch from a CNI‐based to a sirolimus‐based immunosuppressive regimen [30, 31, 32, 33]. For each study, the number of cSCC cases and corresponding person‐years were extracted to calculate incidence rates. In the TUMORAPA trial reported by Euvrard et al., 64 patients were assigned to the sirolimus group and 56 to the CNI group. The calculated IRR for cSCC was 0.39 with a 95% confidence interval (95% CI) of 0.30–0.51, contributing 33.5% weight to the pooled estimate. Campbell et al. reported results from a trial including 86 patients, with an IRR of 0.51 (95% CI 0.34–0.77) and a study weight of 22.9%. Hoogendijk‐van den Akker et al. conducted a multicenter trial enrolling 155 patients, reporting an IRR of 0.59 (95% CI 0.47–0.74) with a weight of 37.9%. Carroll et al. contributed data from a pilot trial with 32 participants, showing an IRR of 0.92 (95% CI 0.32–2.64) and a weight of 5.7%.

**FIGURE 2:**
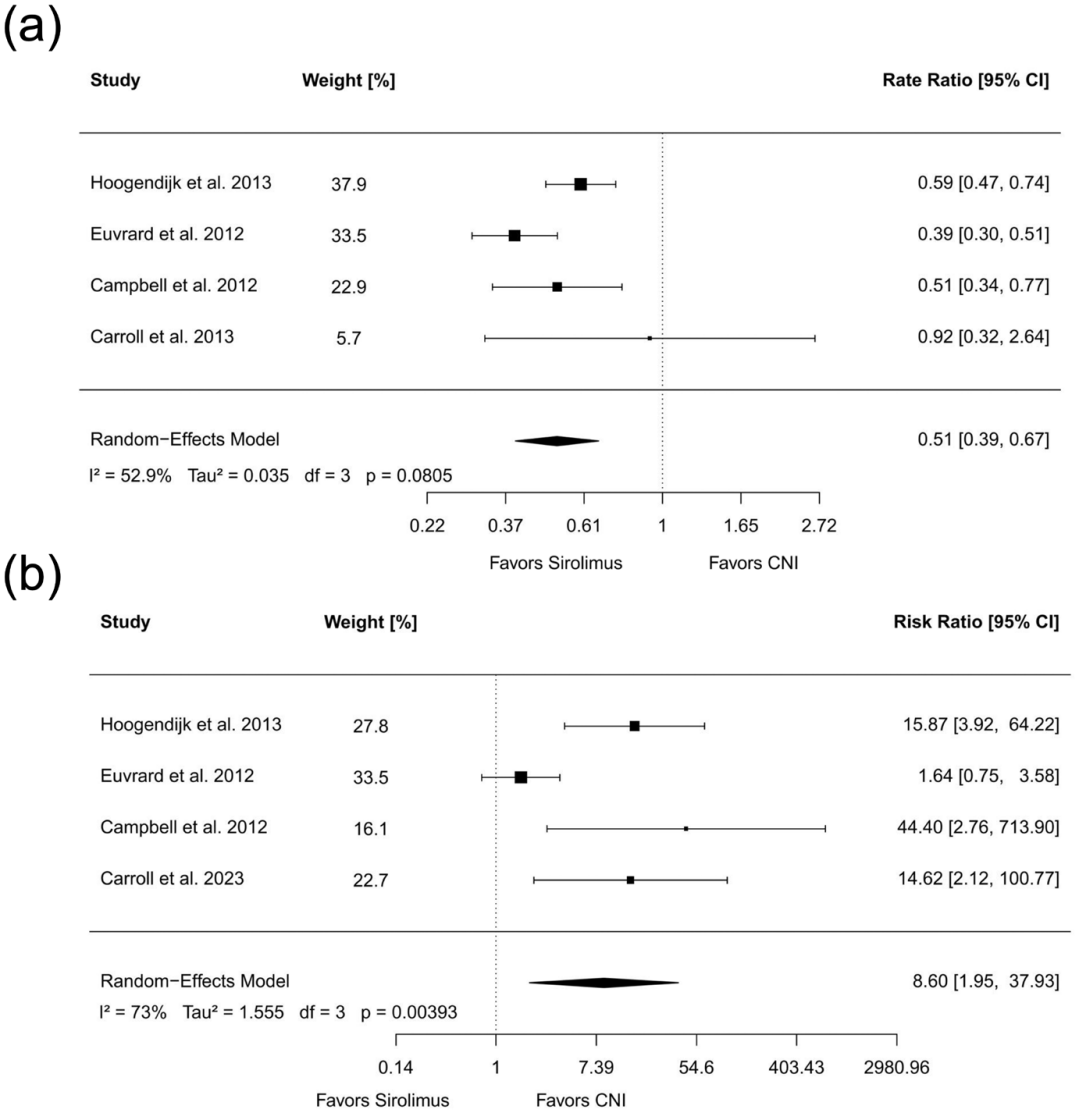
(a) Forest plot showing the incidence rate ratio (IRR) of cutaneous squamous cell carcinoma (cSCC) two years after switching immunosuppressive therapy from calcineurin inhibitors (CNI) to sirolimus. The number of cSCC events and corresponding person‐years were extracted from four randomized controlled trials (RCTs). The pooled estimate showed an approximate 50% reduction in the risk of developing new cSCC over two years (IRR 0.51, 95% CI 0.39–0.67). (b) Forest plot showing the risk ratio (RR) of study discontinuation two years after switching from CNI to sirolimus. Patients who switched to sirolimus had a significantly higher risk of discontinuation (RR 8.60, 95% CI 1.95–37.93) compared to those who remained on CNI. All effect estimates were pooled using a random‐effects model due to anticipated heterogeneity among studies. IRRs incorporate person‐time and capture all events, including multiple tumors in one patient, while accounting for differences in follow‐up duration. RRs reflect the probability of first events and are therefore used for binary adverse‐event outcomes. Hoogendijk et al. refers to the study by Hoogendijk‐van den Akker et al. and was cut due to better clarity of the plot. 95% CI, 95% confidence interval; df, degrees of freedom.

The pooled analysis was conducted using a random‐effects model due to moderate heterogeneity across the included studies (Tau^2^ = 0.035, *I* ^2^ = 52.9%, *p* = 0.0805). The resulting pooled IRR for cSCC was 0.51 with a 95% CI of 0.39–0.67, indicating that patients at high risk for cSCC who switched their immunosuppressive regimen from CNI to sirolimus had a significantly lower risk of developing subsequent cSCC (Figure 2a). Sensitivity analyses were performed by sequentially omitting individual studies. These analyses showed that the overall pooled IRR remained consistent.

In addition, Dantal et al. reported results from a five‐year extension of the TUMORAPA trial [34]. In this follow‐up, the proportion of patients who developed new cSCC lesions in the sirolimus group was significantly lower compared to the CNI group, suggesting that the antitumoral effect of sirolimus was maintained at five years: 22% versus 59% (*p* < 0.001). For the pooled quantitative analysis, only the two‐year data from the four studies described above were included, while the longer‐term findings from Dantal et al. were summarized qualitatively to complement the overall evidence on extended follow‐up.

### 3.4 Incidence of Skin Cancers Other Than Squamous Cell Carcinoma

Two studies also reported incidence rates of skin cancers other than squamous cell carcinoma (SCOSCC). In our meta‐analysis, the results were assessed qualitatively due to the low study number and high heterogeneity of the data. In the study by Euvrard et al., incidence rates for SCOSCC were 1.43 (number per patient over two years) in the sirolimus group and 3.73 in the CNI group. In the sirolimus group, 6 of 42 patients (14%) developed at least one basal cell carcinoma (BCC), 3 patients (7%) developed at least one keratoacanthoma (KA), 4 patients (10%) developed Bowen's disease (BD), and 11 patients (26%) had premalignant keratoses (PK). In the CNI group, 12 of 44 patients (27%) developed at least one BCC, 9 patients (20%) developed KA, 6 patients (14%) developed BD, 16 patients (36%) had PK, and one patient each developed Merkel cell carcinoma and adnexal carcinoma. The study by Campbell et al. reported that 14 of 39 patients (36%) in the sirolimus group and 24 of 47 patients (51%) in the CNI group developed at least one new biopsy‐confirmed BCC over a two‐year period.

### 3.5 Study Discontinuation and Adverse Events

All four included RCTs reported data on study discontinuation rates, but only three studies reported adverse events (Table 2) [30, 31, 32]. The extracted RRs for discontinuation ranged widely across studies. Carroll et al. showed a RR of 14.62 (95% CI 2.12–100.77), Campbell et al. showed a RR of 44.40 (95% CI 2.76–713.90), Euvrard et al. showed a RR of 1.64 (95% CI 0.75–3.58), and Hoogendijk‐van den Akker et al. showed a RR of 15.87 (95% CI 3.92–64.22). The pooled analysis using a random‐effects model showed a combined RR for study discontinuation of 8.60 (95% CI 1.95–37.93), indicating that patients who switched their immunosuppressive therapy were at higher risk for study discontinuation compared to patients who remained on CNI (Figure 2b). The heterogeneity among the studies was substantial (*I* ^2^ = 73%, Tau^2^ = 1.555, *p* = 0.00393).

**TABLE 2:** Summary of reported adverse events (AE) and study discontinuation due to AE.

Adverse event	Hoogendijk et al.		Euvrard et al.		Campbell et al.		Carroll et al.		Total			
	Sirolimus	CNI	Sirolimus	CNI	Sirolimus	CNI	Sirolimus	CNI	Sirolimus	CNI	Pooled RR [95% CI]	*Z*‐value (*p*‐value)
Study discontinuation due to AE	29/74 [39.2]	2/81 [2.5]	15/64 [23.4]	8/56 [14.3]	18/39 [46.2]	0/47 [0]	10/13 [76.9]	1/19 [5.3]	72/190 [37.9]	11/203 [5.4]	8.6 [1.95; 37.93]	2.843 (0.0045)
Transplant rejection	1/74 [1.4]	0/81 [0]	0/64 [0]	0/56 [0]	1/39 [2.6]	0/47 [0]	NR	NR	2/177 [1.1.]	0/184 [0]	2.45 [0.35; 17.16]	0.9 (0.368)
Pneumonitis	2/74 [2.7]	0/81 [0]	14/64 [21.9]	1/56 [1.8]	6/39 [15.4]	0/47 [0]	NR	NR	22/177 [12.4]	1/184 [0.5]	10.85 [2.58; 45.69]	3.251 (0.0012)
Edema	9/74 [12.2]	2/81 [2.5]	37/64 [57.8]	16/56 [28.6]	16/39 [41.0]	3/47 [6.4]	NR	NR	62/177 [35.0]	1/184 [0.5]	3.34 [1.47; 7.59]	2.882 (0.004)
Diarrhea	9/74 [12.2]	0/81 [0]	17/64 [26.6]	6/56 [10.7]	16/39 [41.0]	6/47 [12.8]	NR	NR	42/177 [23.7]	12/184 [6.5]	3.09 [1.72; 5.55]	3.767 (0.0002)
Skin rash	6/74 [8.1]	1/81 [1.2]	5/64 [7.8]	0/56 [0]	13/39 [33.3]	1/47 [2.1]	NR	NR	24/177 [13.6]	2/184 [1.1]	10.22 [2.82; 37.09]	3.535 (0.0004)
Acne	3/74 [4.1]	0/81 [0]	28/64 [43.8]	11/56 [19.6]	7/39 [17.9]	1/47 [2.1]	NR	NR	38/177 [21.5]	12/184 [6.5]	3.2 [1.22; 8.4]	2.358 (0.0184)
Dyslipidemia	13/74 [17.6]	3/81 [3.8]	15/64 [23.4]	1/56 [1.8]	5/39 [12.8]	2/47 [4.3]	NR	NR	33/177 [18.6]	6/184 [3.3]	5.02 [2.11; 11.96]	3.644 (0.0003)
Proteinuria	16/74 [21.6]	0/81 [0]	20/64 [31.3]	4/56 [7.1]	7/39 [18.0]	0/47 [0]	NR	NR	43/177 [24.3]	4/184 [2.2]	8.58 [2.16; 34.09]	3.055 (0.0023)
Died	2/74 [2.7]	1/81 [1.2]	1/64 [1.6]	1/56 [1.8]	1/39 [2.6]	1/47 [2.1]	NR	NR	4/177 [2.3]	2/184 [1.1]	1.39 [0.31; 6.25]	0.429 (0.668)

*Note:* A random‐effects meta‐analysis was performed to evaluate the risk of adverse events associated with sirolimus compared to CNI treatment. Effect measure was the pooled risk ratio (RR) for adverse events of the sirolimus group compared to the calcineurin inhibitor (CNI) group. Each field contains the proportion of patients [%] experiencing the specific AE. The *Z*‐value and corresponding *p*‐value test whether the overall pooled effect differs significantly from one. A *p*‐value < 0.05 indicates statistical significance.

Abbreviations: 95% CI, 95% confidence interval; NR, not reported.

Patients who were converted to sirolimus demonstrated significantly higher pooled RRs for pneumonitis (RR 10.85; 95% CI 2.58–45.69), edema (RR 3.34; 95% CI 1.47–7.59), diarrhea (RR 3.09; 95% CI 1.72–5.55), skin rash (RR 10.22; 95% CI 2.82–37.09), acne (RR 3.20; 95% CI 1.22–8.40), dyslipidemia (RR 5.02; 95% CI 2.11–11.96), and proteinuria (RR 8.58; 95% CI 2.16–34.09) compared to patients who remained on a CNI‐based regimen. However, no significant differences were observed in the meta‐analysis with respect to transplant rejection or mortality. The five‐year extension reported by Dantal et al. also showed that the mean number of serious adverse effects per patient decreased from 1.16 during the first 2 years to 0.83 between years 2 and 5, showing a better sirolimus tolerance over time.

### 3.6 Bias Assessment and Certainty of Evidence

**FIGURE 3:**
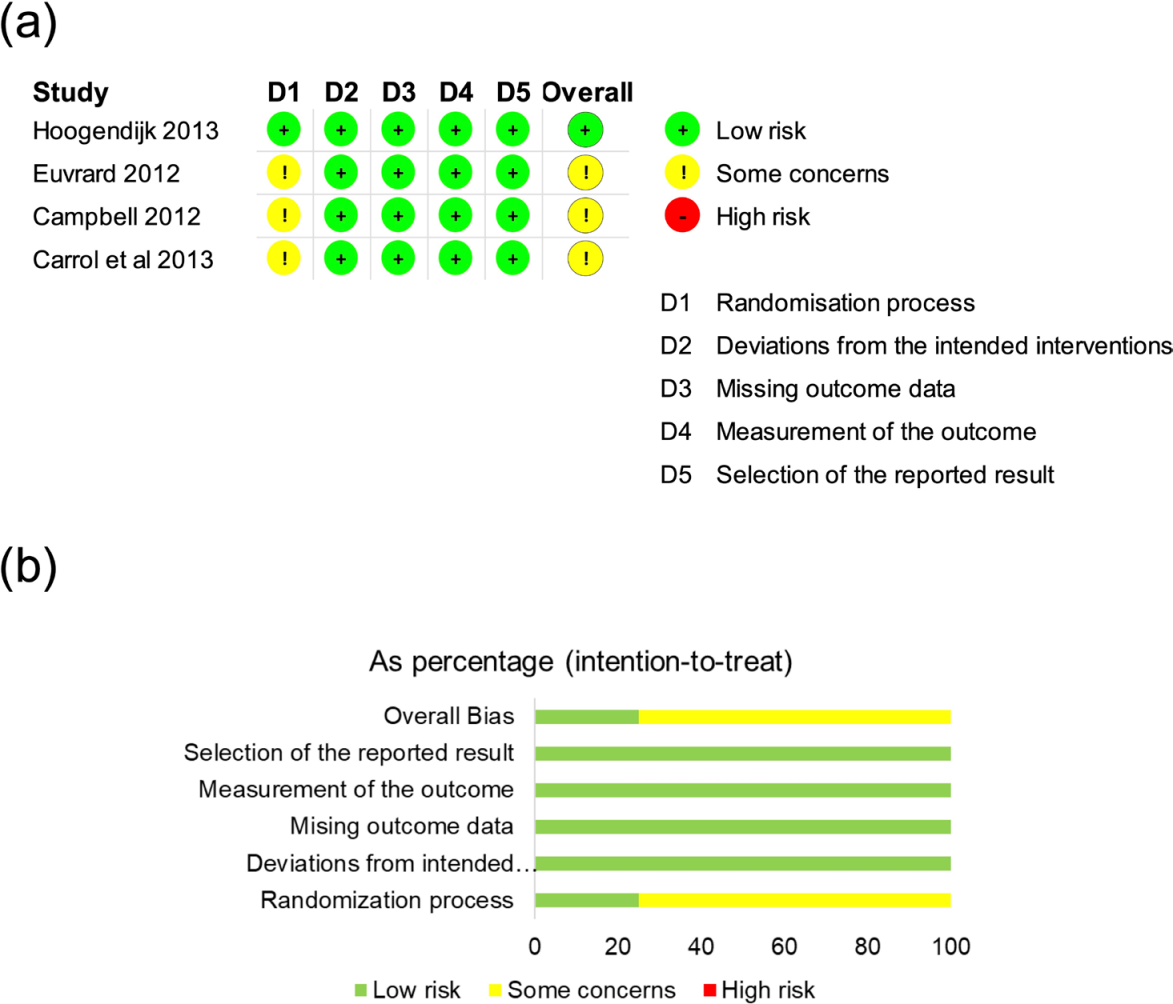
(a) Risk of bias for all outcomes was evaluated by two authors according to the revised Cochrane risk‐of‐bias tool for randomized controlled trials (RoB2). Bias assessment involved five categories: Randomization process (D1), deviations from the intended interventions (D2), missing outcome data (D3), measurement of the outcome (D4) and selection of the reported result (D5). Bias was rated as low risk (green), some concerns (yellow) or high risk (red). (b) The traffic light plot shows the proportion of studies judged as low risk, some concerns, or high risk of bias across the different domains. The figure shows risk of bias assessment for the incidence of cutaneous squamous cell carcinoma after 2 years.

Since fewer than ten studies were included, a funnel plot was not generated, and the possibility of publication bias cannot be ruled out. Regarding the primary outcome (cSCC incidence rate over two years), the studies were well conducted, with low dropout rates, and all performed an intention‐to‐treat analysis. However, three studies did not clearly report whether allocation concealment was adequate, leading to some concerns about the overall randomization process (Figure 3) [31, 32, 33]. Moderate heterogeneity and consistent sensitivity analysis did not lead us to downgrade the certainty of evidence, so we rated the certainty of evidence as high for the outcome cSCC incidence according to GRADE. Regarding adverse events, the risk of bias assessment was comparable overall. However, the study by Campbell et al. did not predefine adverse events and reported only those affecting at least 10% of patients, which could introduce potential selection bias (Figure S1). Due to possible selective reporting and substantial heterogeneity, the certainty of evidence was downgraded by two points. The certainty of evidence for the outcome adverse events was therefore rated as low.

## 4 Discussion

Our results suggest that switching immunosuppressive therapy from CNI to sirolimus can reduce the risk for subsequent cSCCs in KTRs with a history of prior cSCC. Patients who received sirolimus‐based immunosuppression had an approximately 50% reduction in the risk of developing new sSCC over a two‐year period. KTRs with a history of cSCC are at high risk of developing subsequent tumors [11], and these patients also have a significantly higher risk of metastatic progression compared to immunocompetent patients [14], which emphasizes the need for strategies to prevent further cSCCs. This is particularly relevant because treatment options for advanced cSCC in KTRs are severely limited. Immune checkpoint inhibitors (ICI), which are the standard therapy for advanced or metastatic cSCC, can cause graft rejection and subsequent graft loss. When clinically necessary, ICI use may be considered within a shared decision‐making process, carefully balancing antitumor benefit against rejection risk, which is reported to be around 40% [35]. Current literature suggests maintaining immunosuppression at ICI initiation with a dual regimen of an mTOR inhibitor combined with either corticosteroids or a CNI. Any such adjustment must be weighed against the considerable risk of allograft rejection and the potential for tumor progression [18, 36].

Long‐term immunosuppression is a key driver in the development of cSCC, and, in particular, the standard immunosuppressive agent cyclosporin is considered pro‐oncogenic due to both immunosuppression‐dependent and independent mechanisms. Beyond impairing immune surveillance, it directly inhibits DNA repair mechanisms [37, 38], activates oncogenic signaling pathways such as PI3K/AKT/mTOR [39], suppresses p53‐dependent senescence [40] and promotes tumor angiogenesis [41]. In contrast, inhibition of mTOR has distinct anticancer effects, primarily by targeting the PI3K/AKT/mTOR signaling pathway, which is frequently dysregulated in cSCC. This leads to decreased protein synthesis, cell growth, proliferation, induction of cell cycle arrest and apoptosis in cancer cells [42, 43, 44, 45]. Clinically, conversion to mTOR inhibitors has demonstrated efficacy in reducing the incidence of cSCC in the context of primary prevention [8], and even topical sirolimus has shown a preventive effect for cSCC in KTRs [46]. However, routine conversion of all KTRs to mTOR inhibitors is not feasible due to worse tolerability and higher risk of adverse events, including pneumonitis, edema, diarrhea, dyslipidemia and proteinuria. These side effects contribute to a higher discontinuation rate, which is consistent with our findings. Therefore, careful risk stratification is needed to identify patients most likely to benefit from conversion, with a history of prior cSCC serving as a valuable guide for clinical decision‐making.

Our meta‐analysis adds robust evidence from RCTs to support the role of mTOR inhibitors in secondary prevention of cSCC within 2 years after conversion. Importantly, the extended follow‐up data reported by Dantal et al. shows that the protective effect of sirolimus may persist also over the long term with improved tolerability after the first two years [34], highlighting a meaningful gap in long‐term comparative data, as the other RCTs provide shorter follow‐up durations. Remarkably, despite a higher rate of adverse events, our findings do not show a significant increase in allograft rejection or mortality among patients switched to sirolimus, suggesting that this strategy is safe when appropriately monitored. Most complications, including dyslipidemia, cytopenia, ulcers and proteinuria, are dose‐dependent and often reversible with dose reduction or supportive therapy [47, 48, 49, 50]. The KDIGO guideline recommends close monitoring of drug levels and individualized risk assessment to optimize tolerability and minimize toxicity [4]. Early identification and management of metabolic, hematologic and wound‐related complications, such as statin therapy, dose adjustment and avoidance of early post‐transplant initiation in high‐risk patients, are essential to maintaining long‐term efficacy while reducing discontinuation rates [51, 52]. Two RCTs also reported lower rates of non‐cSCC skin cancers, such as BCC, KA and BD, but we did not perform a meta‐analysis due to high heterogeneity and limited study number. In addition to reducing the risk of recurrent skin cancers, secondary prevention strategies such as targeted conversion to mTOR inhibitors may help mitigate cancer‐related fatigue, a common issue in KTRs that can delay or reduce engagement with medical care and negatively affect survival [53]. By limiting recurrent tumor events and associated treatment burden, these strategies may support continued patient participation in follow‐up care, further enhancing long‐term outcomes.

This meta‐analysis has some limitations that should be acknowledged. First, the number of eligible RCTs was limited to four, which restricts the statistical power and precludes a formal assessment of publication bias. We did not identify any unpublished studies, but given the challenges associated with switching immunosuppressive regimes, it is possible that negative or inconclusive trials remain unreported. Second, although all included trials were RCTs, there was moderate heterogeneity among studies. The trials varied in patient populations as well as in baseline risk profiles, such as skin phototype, UV exposure history, or cumulative immunosuppression, and disease severity. Differences in intervention protocols, follow‐up durations and outcome definitions further limited direct comparability, plausibly limiting direct comparability. Third, adverse events were inconsistently reported across studies, with some lacking predefined criteria or comprehensive documentation of toxicity profiles. In particular, the study by Campbell et al. reported only adverse events that affected at least 10% of patients, likely underestimating less frequent or mild adverse events. When we qualitatively accounted for this selective reporting by performing sensitivity assessment, the overall pattern of discontinuation rates and toxicities remained consistent. However, the absolute incidence of milder or rarer events may still be underestimated and readers should interpret the pooled estimate with caution. Fourth, evidence on long‐term outcomes and graft function beyond five years remains scarce.

In summary, this systematic review and meta‐analysis is the first to provide evidence from RCTs that switching immunosuppressive therapy from a CNI‐based to an mTOR inhibitor–based regimen significantly reduces the incidence of subsequent cSCCs in KTRs with a history of cSCC. This effect appears to persist over extended follow‐up and does not seem to increase the risk of allograft rejection or mortality. However, the increased incidence of sirolimus‐related adverse events leads to a higher discontinuation rate, underlining the importance of careful patient selection and risk stratification. In clinical practice, targeted conversion to sirolimus may serve as an effective strategy for secondary prevention in high‐risk KTRs, particularly those with prior cSCC, when supported by appropriate monitoring and toxicity management. Future studies with longer follow‐up and standardized adverse event reporting are needed to refine patient selection criteria and improve long‐term outcomes.

## Funding

The authors have nothing to report.

## Ethics Statement

This systematic review did not require ethical approval as it only involved analysis of previously published data. All included studies were reviewed for ethical compliance and were conducted in accordance with the Declaration of Helsinki and relevant institutional guidelines.

## Conflicts of Interest

The authors declare no conflicts of interest.

## Supporting information


**Figure S1:** (a) Risk of bias for all outcomes was evaluated by two authors according to the revised Cochrane risk‐of‐bias tool for randomized controlled trials (RoB2). Bias assessment involved five categories: Randomization process (D1), deviations from the intended interventions (D2), missing outcome data (D3), measurement of the outcome (D4) and selection of the reported result (D5). Bias was rated as low risk (green), some concerns (yellow) or high risk (red). (b) The traffic light plot shows the proportion of studies judged as low risk, some concerns, or high risk of bias across the different domains. The figure shows risk of bias assessment for adverse events and study discontinuation within 2 years.

**Table S1:** Syntax for systematic search of medical databases MEDLINE, EMBASE and CENTRAL. Two reviewers (YF and OP) independently screened all records identified through Ovid, a web‐based search platform.

**Table S2:** Overview of studies that were excluded during full‐text review. Eight studies were excluded because they focused on primary prevention, one study included patients with a variety of precancerous skin conditions including verrucae, one study lacked a randomized design and one study had no outcome data available due to early study discontinuation.

## Data Availability

The data that support the findings of this study are available from the corresponding author upon reasonable request.
