# Supplementary figures and images for: Sirolimus for Secondary Prevention of Cutaneous Squamous Cell Carcinoma in Kidney Transplant Recipients: A Systematic Review and Meta‐Analysis of Randomized Controlled Trials
Source: Int J Dermatol. 2026 Jan 17;65(5):952–62. doi: 10.1111/ijd.70285 (PMC13067330; doi:10.1111/ijd.70285)

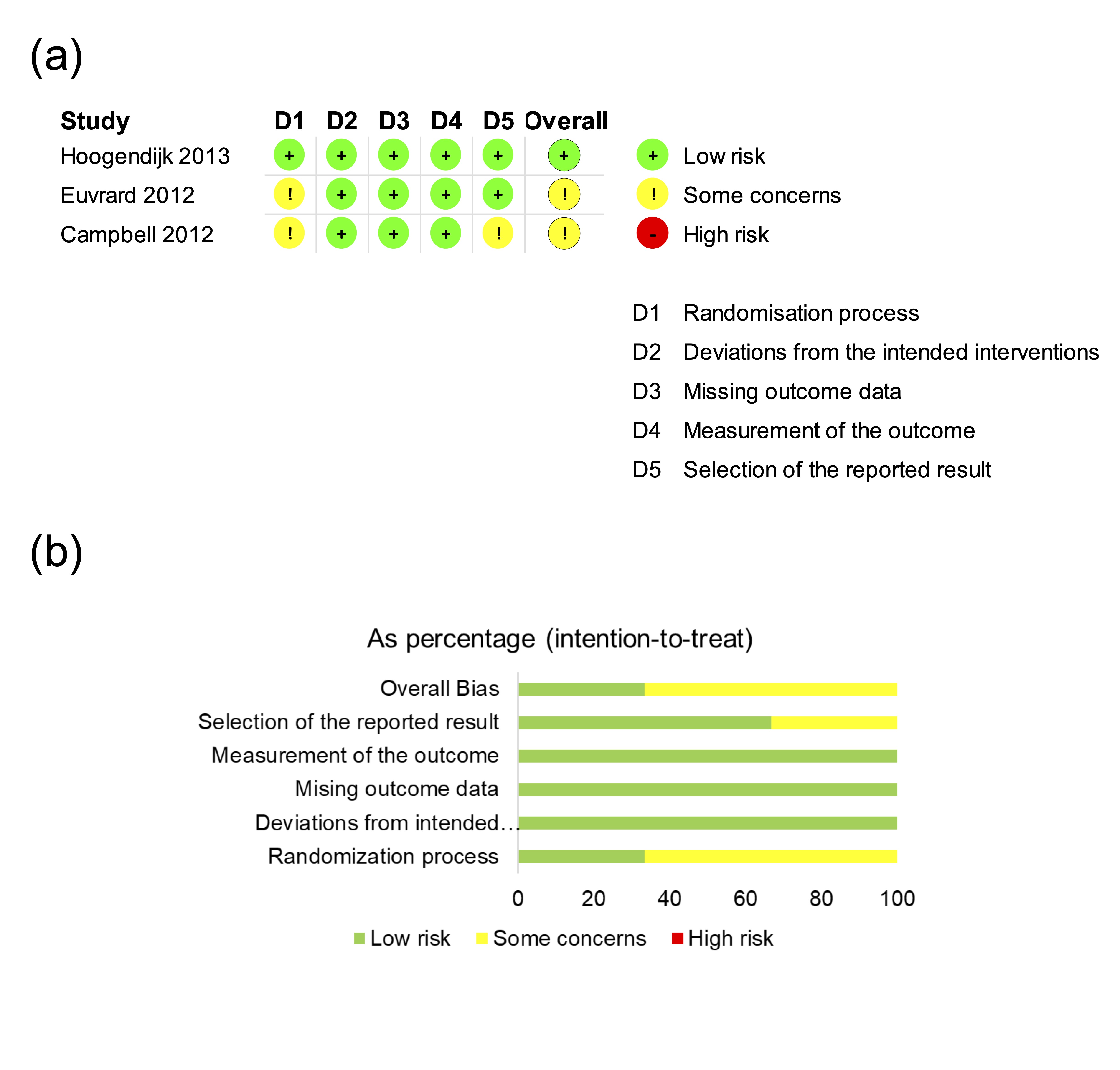

Supplement: Supplementary file 1 — Figure S1: (a) Risk of bias for all outcomes was evaluated by two authors according to the revised Cochrane risk‐of‐bias tool for randomized controlled trials (RoB2). Bias assessment involved five categories: Randomization process (D1), deviations from the intended interventions (D2), missing outcome data (D3), measurement of the outcome (D4) and selection of the reported result (D5). Bias was rated as low risk (green), some concerns (yellow) or high risk (red). (b) The traffic light plot shows the proportion of studies judged as low risk, some concerns, or high risk of bias across the different domains. The figure shows risk of bias assessment for adverse events and study discontinuation within 2 years. [file IJD-65-952-s003.jpg]
