# Supplementary material for: Sirolimus for Secondary Prevention of Cutaneous Squamous Cell Carcinoma in Kidney Transplant Recipients: A Systematic Review and Meta‐Analysis of Randomized Controlled Trials
Source: Int J Dermatol. 2026 Jan 17;65(5):952–62. doi: 10.1111/ijd.70285 (PMC13067330; doi:10.1111/ijd.70285)
Supplement: Supplementary file 2 — Table S1: Syntax for systematic search of medical databases MEDLINE, EMBASE and CENTRAL. Two reviewers (YF and OP) independently screened all records identified through Ovid, a web‐based search platform. [file IJD-65-952-s001.docx]

|  |  |
| --- | --- |
| 1 | Sirolimus |
| 2 | Squamous cell carcinoma |
| 3 | Skin cancer |
| 4 | Malignancy |
| 5 | 2 or 3 or 4 |
| 6 | 1 and 5 |
