# Supplementary material for: Sirolimus for Secondary Prevention of Cutaneous Squamous Cell Carcinoma in Kidney Transplant Recipients: A Systematic Review and Meta‐Analysis of Randomized Controlled Trials
Source: Int J Dermatol. 2026 Jan 17;65(5):952–62. doi: 10.1111/ijd.70285 (PMC13067330; doi:10.1111/ijd.70285)
Supplement: Supplementary file 3 — Table S2: Overview of studies that were excluded during full‐text review. Eight studies were excluded because they focused on primary prevention, one study included patients with a variety of precancerous skin conditions including verrucae, one study lacked a randomized design and one study had no outcome data available due to early study discontinuation. [file IJD-65-952-s002.docx]

| Author | Year | Description | Reason for Exclusion |
| --- | --- | --- | --- |
| Alberu et al.^19^ | 2011 | CONVERT trial. Multicenter study including 830 patients. | Primary prevention |
| Budde et al.^20^ | 2012 | ZEUS study. Multicenter study including 300 patients. | Primary prevention |
| Campistol et al. ^21^ | 2006 | Multicenter study including 430 patients | Primary prevention |
| El-Agroudy et al.^22^ | 2016 | Single-center study including 58 patients | Primary prevention |
| Guba et al ^23^ | 2012 | SMART study. Multicenter study including 140 patients. | Primary prevention |
| Lebranchu et al.^24^ | 2012 | SPIESSER study. Multicenter trial including 145 patients. | Primary prevention |
| Tedesco-Silva et al.^25^ | 2016 | Multicenter study including 254 patients. | Primary prevention |
| Ying et al.^26^ | 2018 | Multicenter study including 279 patients. | Primary prevention |
| Salgo et al.^27^ | 2010 | Single-center study including 44 patients. | Study did not distinguish between cSCC and other skin lesions, including verrucae |
| Tessmer et al.^28^ | 2006 | Single-center study including 24 patients. | No randomized design |
| NCT00866684^29^ | 2007 | Multicenter study. Not completed due to insufficient patient recruitment | No outcome data |
